# An integrated strategy by using target tissue metabolomics biomarkers as pharmacodynamic surrogate indices to screen antipyretic components of Qingkaikling injection

**DOI:** 10.1038/s41598-017-05812-0

**Published:** 2017-07-24

**Authors:** Zhixin Zhang, Fang Lu, Haiyu Liu, Huizhen Zhao, Yuehong Liu, Shuang Fu, Meiling Wang, Ziye Xie, Honghong Yu, Zhenghai Huang, Yanling Zhang, Xiaoyan Gao

**Affiliations:** 1 School of Chinese Material Medica, Beijing University of Chinese Medicine, South of Wangjing Middle Ring Road, Chaoyang District, Beijing, 100102 P.R. China; 2 Key Laboratory of TCM Foundation and New Drug Research, School of Chinese Material Medica, Beijing University of Chinese Medicine, South of Wangjing Middle Ring Road, Chaoyang District, Beijing, 100102 P.R. China

## Abstract

Traditional Chinese medicine (TCM) treatment can be valuable therapeutic strategies. However, the active components and action mechanisms that account for its therapeutic effects remain elusive. Based on the hypothesis that the components of a formula which exert effect would be measurable in target tissue, a target tissue metabolomics-based strategy was proposed for screening of antipyretic components in Qingkaikling injection (QKLI). First, we detected the components of QKLI which could reach its target tissue (hypothalamus) by determining the hypothalamus microdialysate and discovered that only baicalin and geniposide could be detected. Then, by conducting hypothalamus metabolomics studies, 14 metabolites were screened as the potential biomarkers that related to the antipyretic mechanisms of QKLI and were used as its pharmacodynamic surrogate indices. Subsequently, the dynamic concentration of baicalin and geniposide in hypothalamus microdialysates and biomarkers in hypothalamus were measured and correlated with each other. The results indicated that only baicalin shown a good correlation with these biomarkers. Finally, a network pharmacology approach was established to validate the antipyretic activity of baicalin and the results elucidated its antipyretic mechanisms as well. The integrated strategy proposed here provided a powerful means for identifying active components and mechanisms contributing to pharmacological effects of TCM.

## Introduction

Traditional Chinese Medicine (TCM), using several herbs in combination which called formulas, has been recognized as a representative of complementary and alternative medicine^1^. Though has been applied in clinical for a long history, the active components and action mechanisms that account for its therapeutic effects have not been fully explained, which seriously hinders the process for it integration into the modern health-care system^2, 3^. In recent years, although there have been a considerable number of researches into TCM, these traditional strategies were mostly focused on chemical isolation combined with biological activity screening and these processes were time-consuming, labour intensive and also incomprehensive^4^. How to screen the active components in TCM and clarify their precise therapeutic mechanisms is still the most challenging task currently^5^. Thus, the development of a novel strategy for screening the active components and elucidating the mechanisms that account for TCM therapeutic effects is extremely crucial.

There is a general pharmacological screening strategy for TCM which based on the hypothesis that the active components that account for a certain therapeutic effects should appear in the target tissues with appropriate concentrations after its administration^6, 7^. Thus, it is of significance to analyze the components whose content level can be achieved in the target tissue for it is suggesting that these components might exert effect at these tissue sites. As is known to all, the TCM exert effect should be regarded as a process in which the active components interact with our biological system, including the metabolome, proteome, and genome. When the active components in TCM enter into the target tissue, significant changes will occur in its endogenous metabolite compositions in a time-dependent manner^8^. Thus, the central hypothesis of our study is that the absorption and biodegradation of the TCM active components will result in a time-dependent alteration in the metabolic profile in target tissue. Metabolomics can be defined as a comprehensive analytical approach for the study of the small endogenous metabolite in a biological system and to discover the metabolite biomarkers and related biological pathways^9^. Here, to determine which components in TCM contributed to the therapeutic effects, a target tissue metabolomics analysis was applied to identify the metabolite biomarkers that related to the therapeutic mechanisms of TCM, and then these biomarkers were used as the pharmacodynamic surrogate indices for the active components of TCM screening.

Here we present, for the first time, the target tissue metabolomics biomarkers that can be used as the pharmacodynamic surrogate indices to screen the active components and elucidate the mechanisms that account for TCM therapeutic effects. We developed an integrated strategy that combines ultra performance liquid chromatography-tandem mass spectrometry (UPLC-MS/MS) based *in vivo* behavior evaluation of TCM components, tissue metabolomics, statistical analysis and network pharmacology analysis. We demonstrated the utility of this integrated strategy using Qingkaikling injection (QKLI), an important TCM preparation that is widely used in clinic for the treatment of high fever, upper respiratory inflammation, viral encephalitis, hepatitis, stoke, cerebral thrombosis, tonsillitis and tracheitis^10, 11^. Although the antipyretic effect of QKLI is obvious, its antipyretic components and antipyretic mechanisms remain unclear.

**Figure 1:**
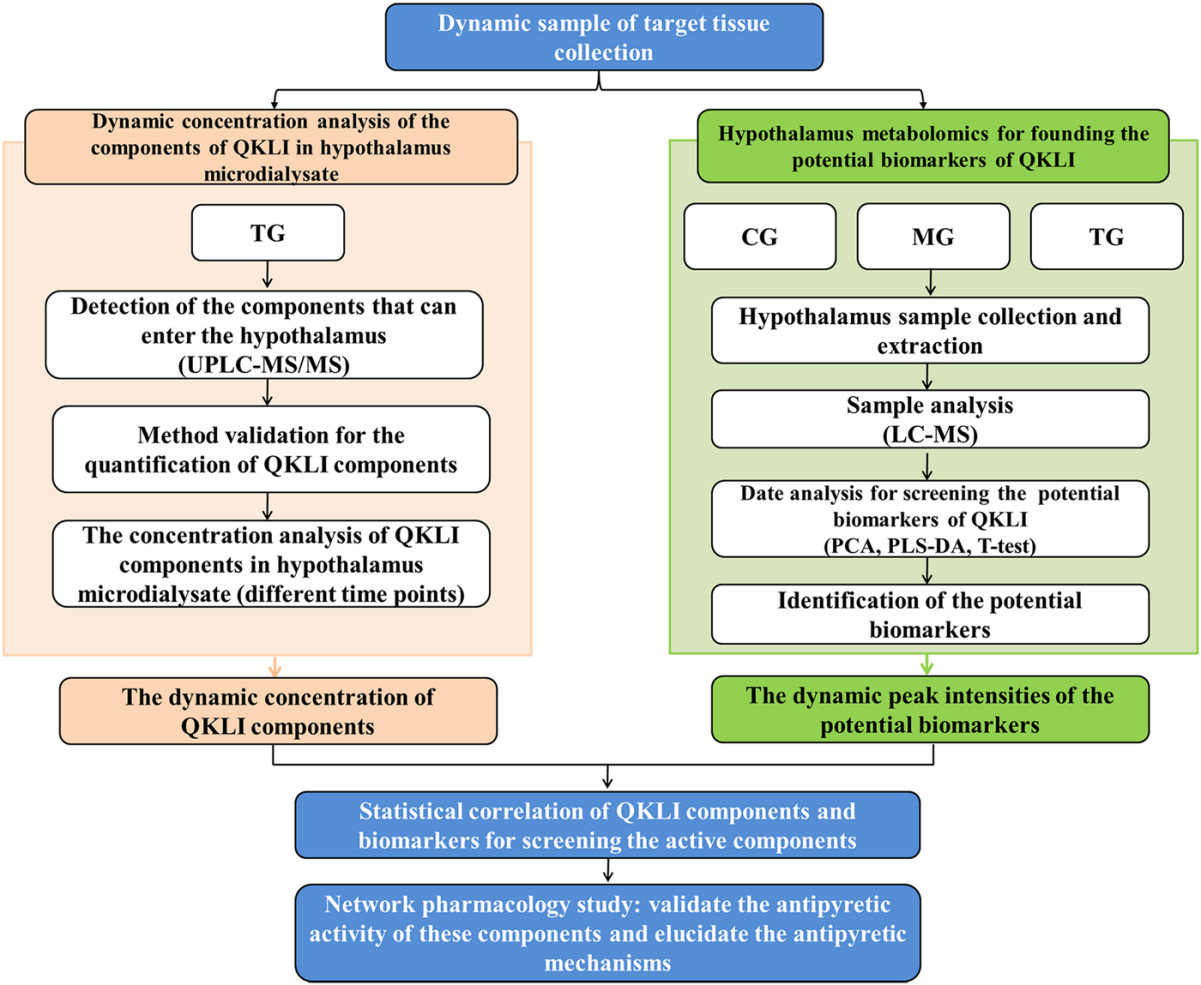
The flow diagram of identifying antipyretic components and mechanisms of QKLI by using target tissue metabolomics biomarkers as pharmacodynamic surrogate indices.

The overall scheme of the proposed strategy is shown in Fig. 1. We first detected the components of QKLI which could reach its target tissue (hypothalamus) by determining the hypothalamus microdialysate of pyrexia model group treated by QKLI (TG) rats. Samples were collected at different time points and the dynamic concentrations of these components were analyzed by a UPLC-MS/MS analytical method. Then, hypothalamus metabolomics analysis was conducted among the control group (CG), pyrexia model group (MG) and TG rats, and the potential biomarkers that related to the antipyretic mechanisms of QKLI were screened and identified through multivariate and univariate statistical analysis. Next, these biomarkers were used as the pharmacodynamic surrogate indices of QKLI, the dynamic peak intensities of them in TG rats were analysis and a correlation analysis was performed between these biomarkers and the components originated from QKLI in hypothalamus microdialysate. The corresponding correlation coefficient described the degree of correlation between them, and those components which have a high degree of correlation with biomarkers were identified as the potential active components that account for the antipyretic effects of QKLI. A network pharmacology approach was further established to validate the antipyretic activity of these components and the results elucidated the antipyretic mechanisms as well. This integrated strategy may provide a new way for screening the active components and elucidating the therapeutic mechanisms of TCM and other complex systems.

## Results

### Determination of the components that can reach the target tissue by hypothalamus microdialysis study

In our previous study, a simple, rapid and sensitive UPLC-MS/MS method was developed for the simultaneous determination of chlorogenic acid, neochlorogenic acid, baicalin, geniposide, cholic acid and hyodeoxycholic acid in rat plasma. Simultaneously, this validated method was successfully applied to the pharmacokinetic study of the six analytes in rats following an intravenous administration of QKLI^12^. To clarify the antipyretic components of QKLI, based on the previous works, a hypothalamus microdialysis study was introduced here to detect the components of QKLI that could pass through the blood brain barrier (BBB) and reach the hypothalamus. The detailed information of the microdialysis procedure, microdialysis probe recovery and method validation was shown in Supplementary Information. The results revealed that only baicalin and geniposide could be detected in the hypothalamic dialysate after intravenous administration of QKLI (Supplementary Figure S1).

**Figure 2:**
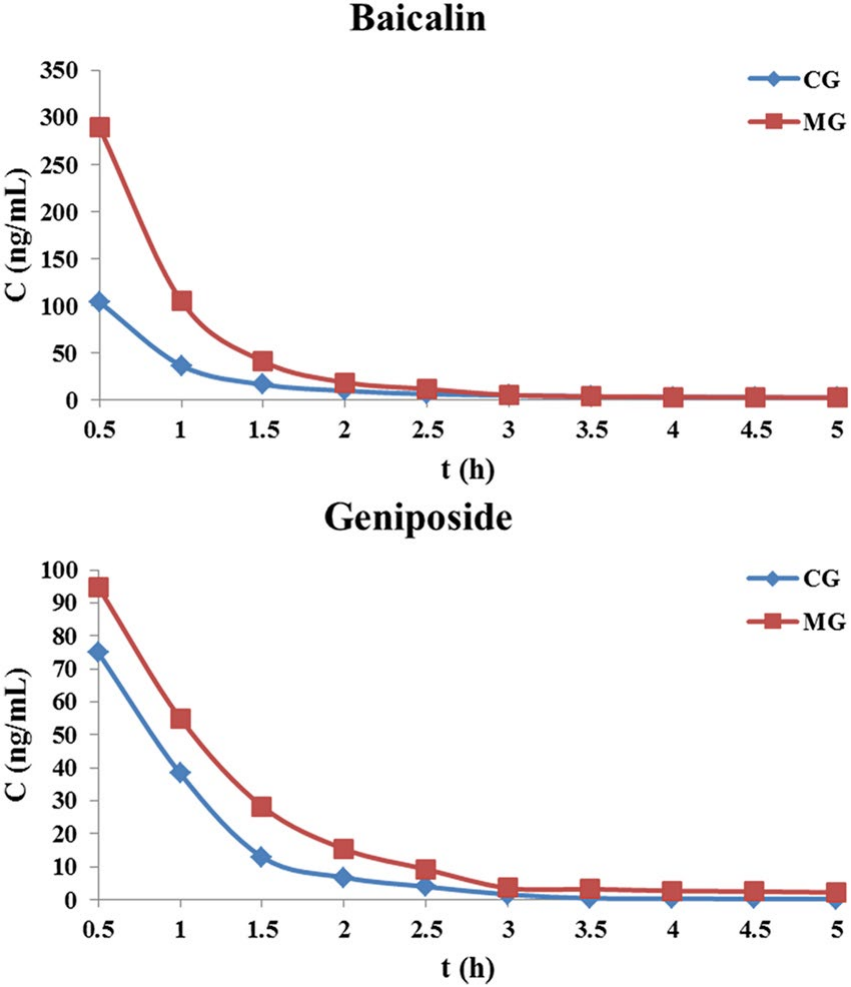
Hypothalamus microdialysis concentration-time profiles of baicalin and geniposidein in CG and MG rats after intravenous administration of QKLI.

We next compared the pharmacokinetic behaviors of baicalin and geniposide in QKLI between the CG and MG rats. Figure 2 shown the mean (n = 6) concentration–time profiles of the two analytes in the two groups after intravenous administration of QKLI, and the main pharmacokinetic parameters were summarized in Table 1. In both groups, baicalin and geniposide could quickly penetrate the BBB and reached the hypothalamus, and the results demonstrated that there were significant differences (*p* < 0.05) in pharmacokinetic parameters including C_max_, AUC_0-t_ and AUC_0-∞_ for the two analytes between two groups. A remarkable increase (*p* < 0.05) in the value of C_max_, AUC_0-t_ and AUC_0-∞_ were observed for the two analytes in MG after intravenous administration of QKLI compared to CG, which pointed out that the absorption of baicalin and geniposide was increased in MG than CG. This interesting phenomenon suggested that baicalin and geniposide might be the antipyretic components of QKLI and prompted us to examine the relationships between baicalin, geniposide and the antipyretic effect of QKLI in hypothalamus.

**Table 1:** Pharmacokinetic parameters of baicalin and geniposide in CG and MG rats after intravenous administration of QKLI.

Pharmacokinetic parameters	Baicalin		Geniposide	
	CG	MG	CG	MG
t_1/2_ (h)	2.131 ± 1.389	1.541 ± 0.903	1.510 ± 1.510	1.229 ± 0.665
t_max_ (h)	0.5	0.5	0.5	0.5
C_max_ (ng/mL)	97.372 ± 20.081	215.313 ± 74.120**	50.348 ± 23.899	70.081 ± 24.863*
AUC_0-t_ (h·ng/mL)	83.114 ± 30.364	195.187 ± 73.973**	42.553 ± 30.721	70.324 ± 30.291*
AUC_0-∞_ (h·ng/mL)	89.644 ± 39.257	212.450 ± 102.092**	45.019 ± 36.711	72.959 ± 30.230*
MRT_0-t_ (h)	0.979 ± 0.136	1.047 ± 0.307	0.894 ± 0.123	1.125 ± 0.253
MRT_0-∞_ (h)	1.449 ± 0.616	1.434 ± 0.968	1.131 ± 0.534	1.363 ± 0.420

*p < 0.05, **p < 0.01, compared with CG rats.

### Using target tissue metabolomics biomarkers as pharmacodynamic surrogate indices for clarifying the antipyretic active components of QKLI

We next sought to investigate the relationships between baicalin, geniposide and the antipyretic effect of QKLI in hypothalamus from yeast-induced pyrexia rats. Usually, the rats’ rectal temperature was used as the pharmacodynamic index for evaluating the antipyretic effect of QKLI, however, for the hysteresis and rough of temperature as pharmacodynamic index, it is hard to make a directly correlation with the dynamic concentration of drug. Therefore, in this section, a metabolomics strategy was conducted here to found the biomarkers in hypothalamus that related to the antipyretic mechanisms of QKLI, and these biomarkers were used as the pharmacodynamics surrogate indices for screening the antipyretic components of QKLI. The hypothalamus microdialysates and hypothalamus samples of TG rats were collected at each time point of 0.5 h, 1 h, 1.5 h, 2 h, 2.5 h, 3 h, 3.5 h, 4 h, 4.5 h and 5 h after QKLI administration, then, the dynamic concentration of baicalin and geniposide in hypothalamus microdialysates and the metabolomics biomarkers in hypothalamus were measured and correlated with each other.

#### Hypothalamus metabolomics study found the potential biomarkers related to antipyretic mechanism of QKLI

The untargeted metabolic profiling of hypothalamus samples was conducted by a high performance liquid chromatography coupled with linear ion trap-orbitrap mass spectrometry (HPLC-LTQ/Orbitrap MS) method. The detailed information of the HPLC-LTQ/Orbitrap MS validation was shown in Supplementary Information. Data acquisition was performed in both positive and negative ion mode. The typical total ion chromatograms (TIC) from CG, MG, and TG rats at different sampling time points (0.5 h, 1 h, 1.5 h, 2 h, 2.5 h, 3 h, 3.5 h, 4 h, 4.5 h and 5 h) were shown in Supplementary Figures S2. The raw mass data were transformed using TransOmics^TM^ and converted into three-dimensional data: sample name, *t*_R_-*m/z* pair and normalised ion intensity for further analysis.

**Figure 3:**
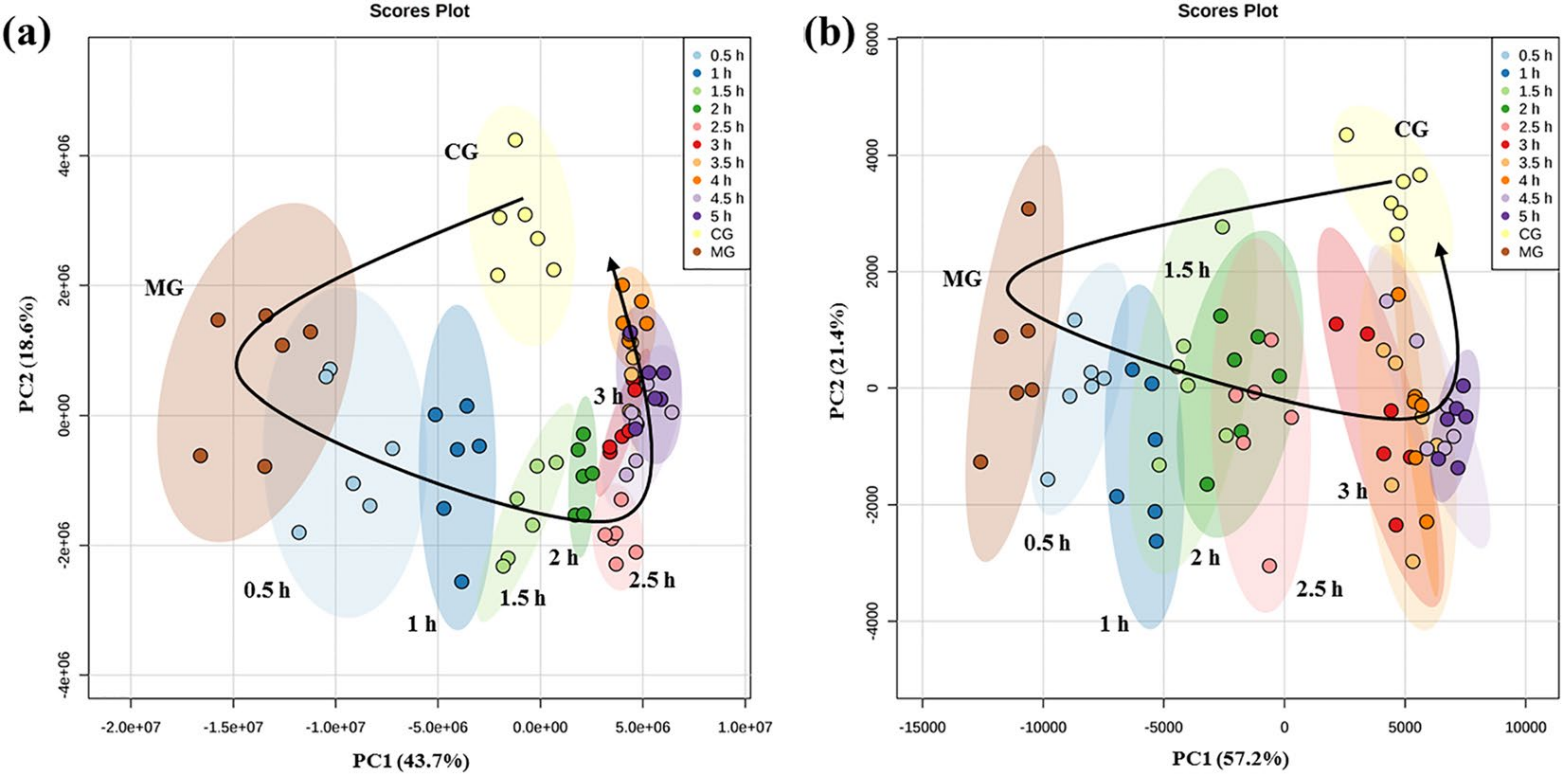
PCA scores plot among CG, MG, and TG rats at different sampling time points. The plot showed a time dependent trajectory of hypothalamus metabolites which clustered at different spatial positions. (**a**) At positive ion mode. (**b**) At negative ion mode.

A principal components analysis (PCA) method was used here for unsupervised multivariate analysis to visualize the hypothalamus metabolic profiling differences between these groups. Figure 3 shown the PCA scores plots among CG, MG, and TG rats at different sampling time points, as can be seen from the figure, it showed a time dependent trajectory of hypothalamus metabolites which clustered at different spatial positions. The MG exhibited obvious separation from the CG, which indicated that fever significantly altered the metabolic pattern of hypothalamus endogenous metabolites in rats. Besides, the metabolic profile of TG rats at different sampling time points showed a gradual recovery trend, and after 3 h of QKLI administration, the metabolite profile was close with that of CG rats, suggesting a recovery of hypothalamus metabolites after QKLI treating. Therefore, the TG rats at the time point of 3 h were selected here to screen the antipyretic active components of QKLI.

**Figure 4:**
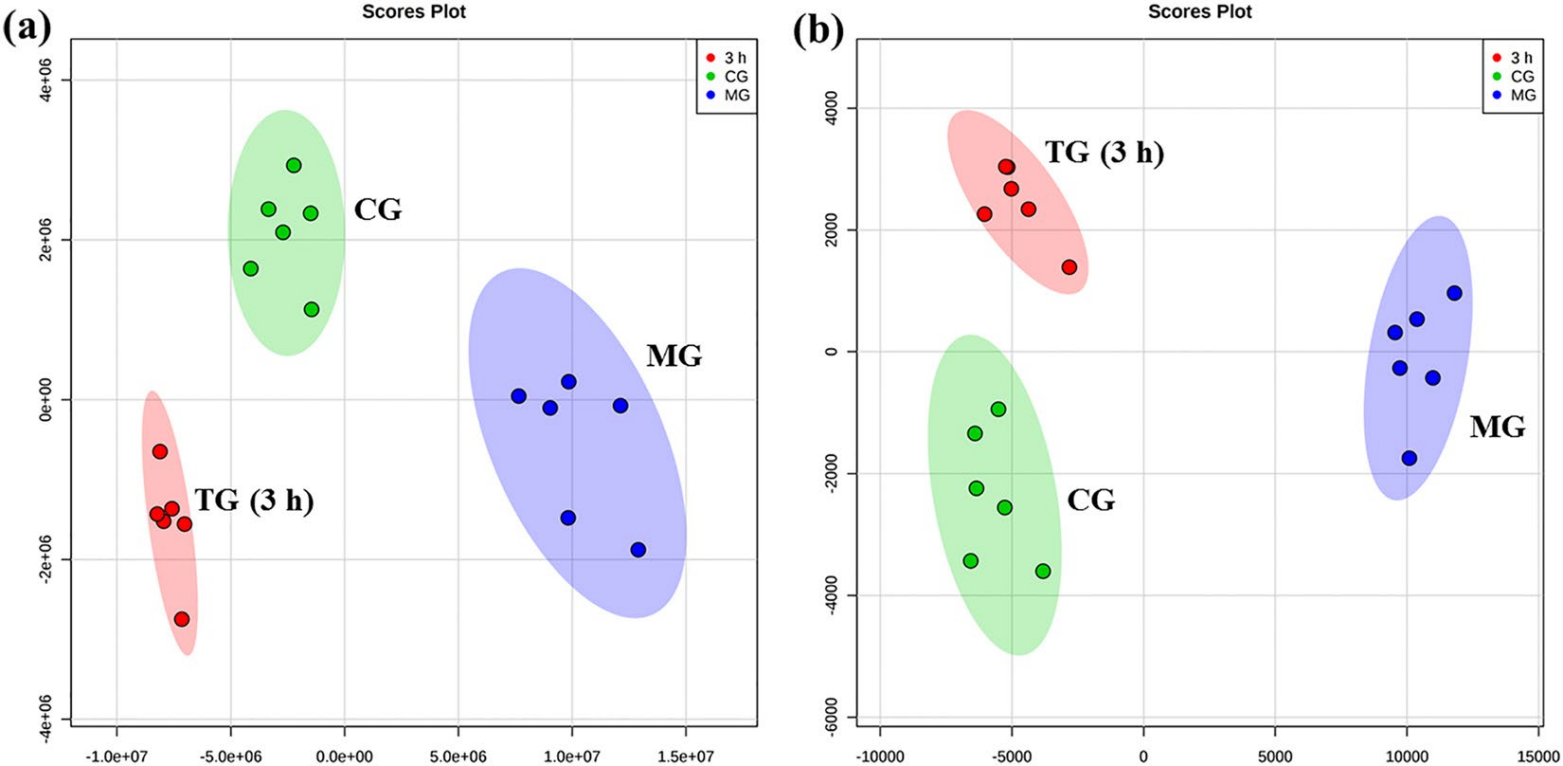
PLS-DA score plot obtained from CG (), MG (), and TG at 3 h (). (**a**) At positive ion mode. (**b**) At negative ion mode.

In order to screen the potential biomarker that related to the antipyretic mechanism of QKLI, a partial least squares-discriminate analysis (PLS-DA) analysis was conducted among CG, MG and TG rats at the time point of 3 h (Fig. 4). The result demonstrated that the metabolites in the MG rats were disturbed by the fever pathological state (MG *vs* CG) and the TG rats have a trend return to normal based on the plots of TG were gradually close to the plots of CG. Based on the PLS-DA analysis, the potential biomarkers were selected for three rules: (1) the VIP (variable importance in the projection) values from the PLS-DA model must be higher than 1; (2) the *T*-test is statistically significant; (3) the compound changing trends of MG and TG must be opposite. According to these rules, 6 endogenous metabolites in positive ion mode and 12 endogenous metabolites in negative ion mode were screened as the potential biomarkers that related to the antipyretic mechanism of QKLI respectively. There were four same metabolites in both positive and negative ion mode. The identification of these biomarkers was conducted by comparing the accurate mass and MS^n^ fragment information extracted by the Xcalibur workstation with those in the biochemical databases, such as METLIN, HMDB and so on. The detailed identification procedure was shown in Supplementary Information. These metabolites with the VIP value, retention time, *m/z* and change trends were shown in Table 2.

**Table 2:** Metabolites selected as biomarkers characterized in hypothalamus profiles and their change trends.

No.	Compounds	*t*_R_ (min)	*m/z*	VIP	Trend in MG^a^	Trend in TG^b^
**Positive ion mode**						
1	Adenosine monophosphate (AMP)	6.99	348.0700	6.4	↑^***^	↓^*^
2	ADP-D-ribose	10.26	560.0785	2.75	↑^***^	↓^***^
3	Guanosine monophosphate (GMP)	10.88	364.0648	3.49	↑^***^	↓^***^
4	Adenosine	11.52	268.1042	1.83	↑^**^	↓
5	Inosinic acid (IMP)	12.22	349.0539	3.17	↑^***^	↓^***^
6	N6-(1,2-dicarboxyethyl)-AMP	17.97	464.0794	2.58	↑^***^	↓^***^
**Negative ion mode**						
1	L-Aspartic acid	2.91	132.0302	1.42	↑^*^	↓^**^
2	5’-CMP	3.47	322.0441	1.21	↑^**^	↓^**^
3	D-Glucuronic acid	4.27	175.0248	4.03	↑^***^	↓^**^
4	Adenosine monophosphate (AMP)	7.09	346.0552	10.3	↑^***^	↓^***^
5	Uridine	9.27	243.0620	2.75	↓^**^	↑^*^
6	Oxidised glutathione	9.61	611.1441	5.32	↑^***^	↓^***^
7	N-acetylaspartylglutamic acid	10.80	303.0831	2.38	↑^*^	↓^*^
8	Guanosine monophosphate (GMP)	10.97	362.0502	4.64	↑^***^	↓^***^
9	ADP	11.23	426.0220	1.75	↑^*^	↓^***^
10	Inosinic acid (IMP)	12.33	347.0388	7.34	↑^**^	↓^***^
11	UDP-D-galactose	17.01	565.0474	3.27	↑^**^	↓^***^
12	N6-(1,2-dicarboxyethyl)-AMP	18.10	462.0667	4.97	↑^***^	↓^***^

The levels of potential biomarkers were labeled with (↓) downregulated and (↑) upregulated (^*^ *P* < 0.05; ^**^ *P* < 0.01; ^***^ *P* < 0.001; one-way ANOVA followed by independent samples ***t***-test).

^a^Change trend compared with CG.

^b^Change trend compared with MG.

#### Dynamic concentration of baicalin, geniposide and biomarkers in target tissue

**Figure 5:**
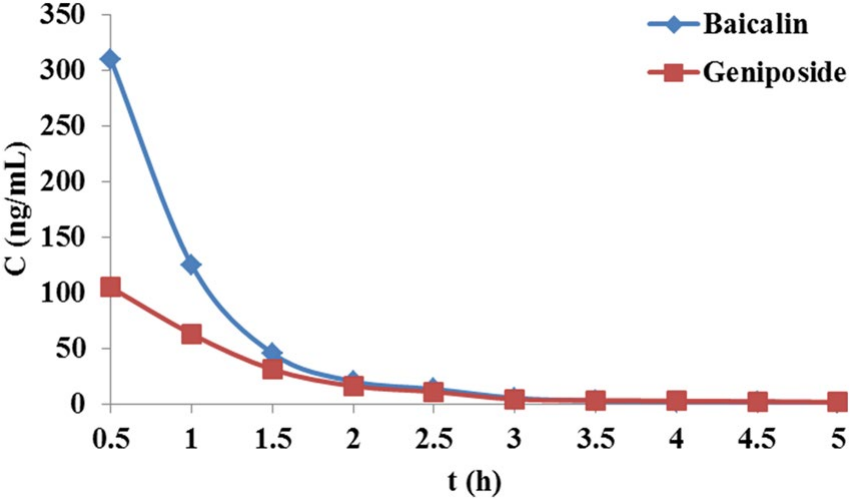
Hypothalamus microdialysates concentration−time courses of baicalin and geniposide in TG rats after intravenous administration of QKLI.

To use the metabolomics biomarkers as pharmacodynamic surrogate indices for clarifying the antipyretic active components of QKLI, the different time point samples of TG rats were collected after QKLI administration. The dynamic concentration of baicalin and geniposide in hypothalamus microdialysates were determinated and their concentration–time courses were illustrated in Fig. 5. Similar to the results of CG and MG rats described above, baicalin and geniposide could quickly get to the hypothalamus and reached their maximum levels at 0.5 h after intravenous administration. Correspondingly, the dynamic peak intensities of the 14 biomarkers in hypothalamus were analyzed and then correlation analyses were conducted between them and the dynamic concentration of baicalin and geniposide.

#### Correlation analysis between baicalin, geniposide and metabolomics biomarkers

To determine whether baicalin or geniposide has a relationship with the antipyretic effect of QKLI, a correlation analysis was established between these two components and the 14 metabolomics biomarkers of QKLI. The correlation coefficient between the dynamic concentration of baicalin, geniposide and the biomarkers were summarized in Table 3. Linear correlation regression revealed that ADP-D-ribose (R^2^ = 0.745), N6-(1,2-dicarboxyethyl)-AMP (R^2^ = 0.727), GMP (R^2^ = 0.702) and IMP (R^2^ = 0.673) in the positive ion mode and N6-(1,2-dicarboxyethyl)-AMP (R^2^ = 0.795), GMP (R^2^ = 0.709), UDP-D-galactose (R^2^ = 0.670) in the negative ion mode were highly correlated with baicalin while geniposide did not shown statistically significant correlation with any of these biomarkers (R^2^ < 0.6). Therefore, we hypothesised that baicalin might play the important roles in the antipyretic effect of QKLI.

**Table 3:** The correlation coefficients between baicalin, geniposide and potential biomarkers.

No.	Compounds	Baicalin (R^2^)	Geniposide (R^2^)
**Positive ion mode**			
1	Adenosine monophosphate (AMP)	0.476	0.308
2	ADP-D-ribose	0.745	0.362
3	Guanosine monophosphate (GMP)	0.702	0.387
4	Adenosine	0.547	0.328
5	Inosinic acid (IMP)	0.673	0.024
6	N6-(1,2-dicarboxyethyl)-AMP	0.727	0.373
**Negative ion mode**			
1	L-Aspartic acid	0.531	0.260
2	5′-CMP	0.402	0.305
3	D-Glucuronic acid	0.473	0.268
4	Adenosine monophosphate (AMP)	0.528	0.362
5	Uridine	0.473	0.161
6	Oxidised glutathione	0.526	0.077
7	N-acetylaspartylglutamic acid	0.540	0.029
8	Guanosine monophosphate (GMP)	0.709	0.089
9	ADP	0.433	0.404
10	Inosinic acid (IMP)	0.516	0.008
11	UDP-D-galactose	0.670	0.546
12	N6-(1,2-dicarboxyethyl)-AMP	0.795	0.157

### A network pharmacology study deciphering the antipyretic effect of baicalin

**Figure 6:**
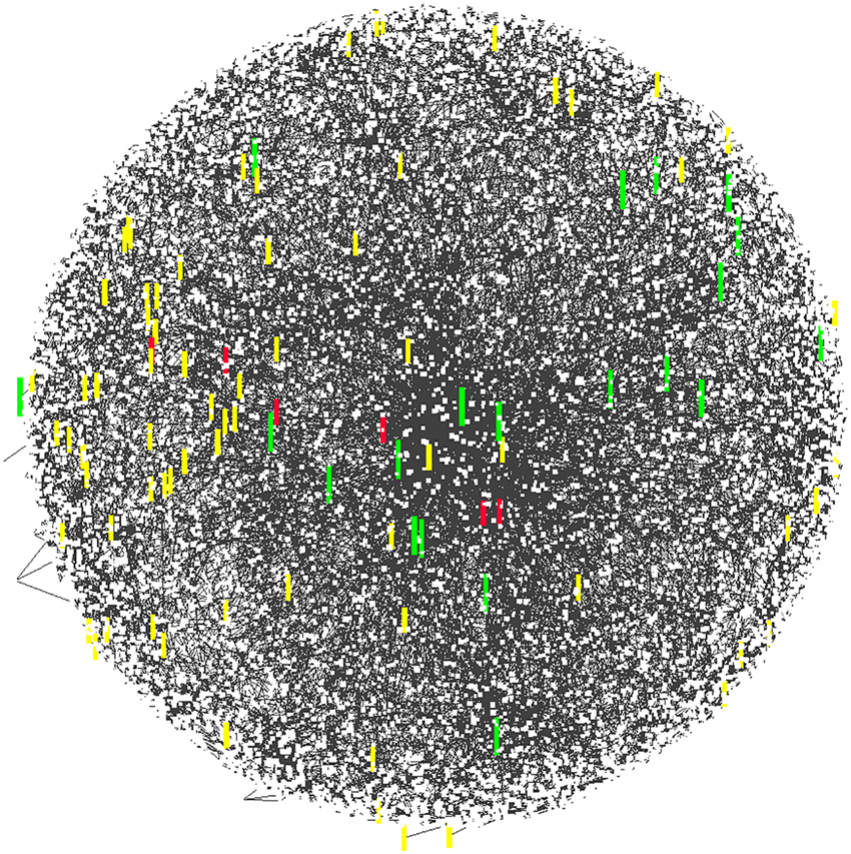
The metabolic network of baicalin (Yellow indicates the baicalin-related targets, green indicates the verified biomarkers and red indicates the fever-related molecules).

It was shown that five potential biomarkers of QKLI in hypothalamus had strong statistical correlations with baicalin. In order to further validate the antipyretic activity of baicalin from the view of molecular level, a novel network pharmacology study was established. Here, eleven baicalin-related targets and fourteen biomarkers-related targets were derived from STITCH database. Then, the two sets of targets, five biomarkers mentioned above and five fever-related molecules (prostaglandin E2 (PGE2), cyclin adenosine monophosphate (cAMP), nitric oxide (NO), arachidonic acid (AA), and prostaglandin F2alpha (PGF2α)), which collected from related references^13–15^, were used as search terms to collect the corresponding metabolic pathways respectively. Figure 6 shown the final metabolism network of baicalin that obtained by merging these four networks together and it was used to deduce the potential antipyretic mechanism of baicalin at molecular level (the detailed information for the network constructing was shown in Supplementary Information). Based on the constructed metabolism network of baicalin, here, two baicalin-related targets, Caspase-3 (CASP3) and Hypoxia-inducible factor 1-alpha (HIF-1α), were chosen as examples to illuminate the potential antipyretic mechanism of baicalin.

#### Antipyretic mechanism of CASP3

**Figure 7:**
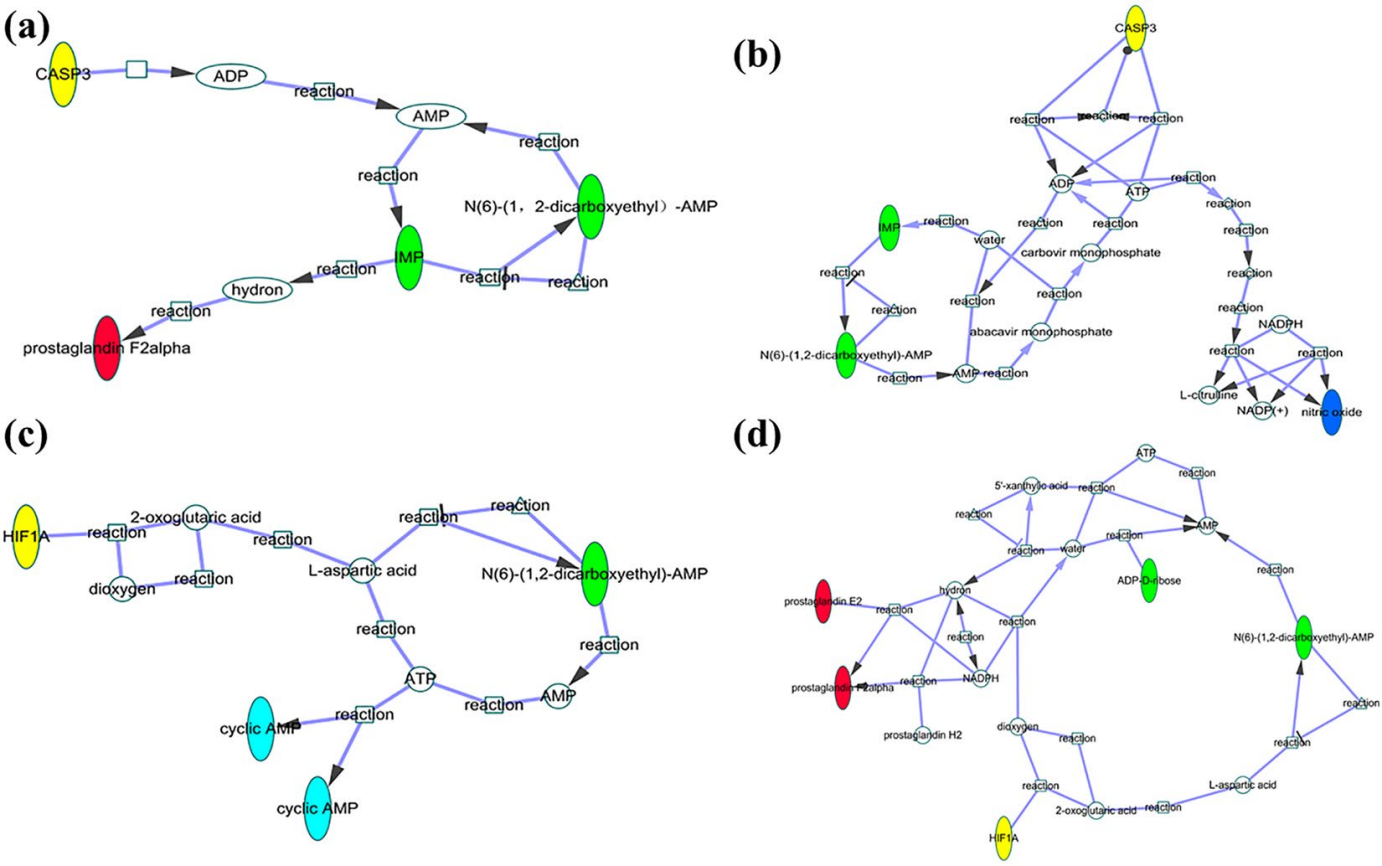
The metabolic pathway of baicalin-related targets relate to verified biomarkers and fever-related molecules. (**a**) The metabolic pathway of baicalin-related target CASP3 relate to fever-related molecule PGF2α (Yellow indicates CASP3, green indicates IMP and N6-(1,2-dicarboxyethyl)-AMP and red indicates PGF2α); (**b**) Metabolic pathway of baicalin-related target CASP3 relate to fever-related molecule NO (Yellow indicates CASP3, green indicates IMP and N6-(1,2-dicarboxyethyl)-AMP and blue indicates fever-related molecule NO); (**c**) The metabolic pathway of baicalin-related target HIF-1α relate to fever-related small molecule cAMP (Yellow indicates HIF-1α, green indicates N6-(1,2-dicarboxyethyl)-AMP and azure indicates cAMP); (**d**) Metabolic pathway of baicalin-related target HIF-1α to fever-related molecule PGE2, PGF2α (Yellow indicates HIF-1α, green indicates IMP and N6-(1,2-dicarboxyethyl)-AMP and red indicates PGE2, PGF2α).

Two fever-related small molecules, PGF2α and NO, can be regulated by CASP3 through a series of metabolic pathways. Specific analyses are as follows: (1) regulation of PGF2α: Fig. 7a shows that, CASP3, one of the baicalin-related targets, can disturb PGF2α through cascade reactions. In these reactions, two biomarkers of QKLI, N6-(1,2-dicarboxyethyl)-AMP and IMP, are synthesized by ADP, ATP, and AMP. This process belongs to purine metabolism, which is related to yeast-induced pyrexia based on recent study^16^. In addition, PGF2α, which is one of the major mediators of fever and can induce pyrexia directly, is produced by IMP in this process^14^; (2) regulation of NO: As shown in Fig. 7b, CASP3 can regulate the synthesis of NO through energy metabolism pathway. Recently study has indicated that NO is connected with pyrexia and inflammation^15^. In conclusion, baicalin can regulate fever-related molecules PGF2α and NO by targeting on CASP3 and then plays antipyretic effect.

#### Antipyretic mechanism of HIF-1α

HIF-1α is another important target of baicalin, which can regulate the fever-related small molecules, such as cAMP, PGE2, and PGF2α through a strand of metabolic pathways. Specific analyses are as follows: (1) regulation of cAMP: In Fig. 7c, HIF-1α can regulate the synthesis of L-aspartic acid through metabolic pathway. Then, on one hand, L-aspartic acid, IMP, and ATP (GTP) can generate AMP through purine metabolism and cyclic adenosine monophosphate (cAMP) is a form of AMP. On the other hand, L-aspartic acid and AMP can produce ATP through different pathways, respectively. ATP can be catalyzed by adenylyl cyclases to generate the second messenger cAMP, which is an important centric febrile mediator^17^. cAMP can cause the release of intracellular Ca^2+^ by targeting on the calcium channel^18^. Then, the excessive content of Ca^2+^ in the brain can in turn increase the concentration of cAMP. The above metabolic pathways are involved in the process of causing fever^19–21^. In conclusion, HIF-1α can connect to cAMP through series reactions, which shows that HIF-1α can regulate fever through these pathways; (2) regulation of PGE2 and PGF2α: as shown in Fig. 7d, HIF-1α can regulate the synthesis of L-aspartic acid through cascade reactions and then L-aspartic acid can involve in the production of ATP through energy metabolism and purine metabolism. ATP reacts with glutamate to generate the intermediate products of guanylate acid anabolism-xanthine nucleotides. This process provides raw materials for the production of AA and then AA further generates PGE2 and PGF2α. AA participates in quantities of inflammation-related metabolic pathways and PGE2 and PGF2α can induce fever directly. Furthermore, PGE2, which can penetrate the BBB, can affect the hypothalamus regions and then induce fever^22^. In conclusion, the above results show that baicalin can regulate fever-related molecules cAMP, PGE2 and PGF2α by targeting on HIF-1α through a train of metabolic pathways.

## Discussion

TCM is widely used and accepted by people around the world for its sound effects in the treatment of numerous diseases. Although lots of research works have been done to identify the material basis for TCM, its exactly therapeutically active components and action mechanisms remain unclear. The lack of efficient methodologies and strategies hinders the modernization process of TCM. In this study, we established an integrated strategy by using target tissue metabolomics biomarkers as pharmacodynamic surrogate indices for identifying the therapeutic active components and mechanisms of TCM. This integrated strategy was applied to screen the antipyretic components and elucidate antipyretic mechanisms of QKLI. Based on our previous study^12^, we first conducted a hypothalamus microdialysis study to detect the components of QKLI that could reach the hypothalamus and compared the pharmacokinetic behaviors of them between the CG and MG rats. Interestingly, the results show that only baicalin and geniposide could be detected and the absorption of them was increased in MG than CG rats. This interesting observation has prompted us to examine the relationships between these two components and the antipyretic effect of QKLI. Next, using hypothalamus metabolomics studies conducted on CG, MG and TG rats, we identified 14 biomarkers that related to the antipyretic mechanisms of QKLI and were used as its pharmacodynamic surrogate indices. The different time point samples of TG rats were collected and the dynamic concentration of baicalin and geniposide in hypothalamus microdialysates and biomarkers in hypothalamus were measured and correlated with each other. The results indicated that five metabolites (ADP-D-ribose, N6-(1,2-dicarboxyethyl)-AMP, GMP, IMP and UDP-D-galactose) of these biomarkers were showed a good correlation with baicalin while geniposide did not show statistically significant correlation with them. Finally, based on the targets of baicalin, potential biomarkers that were correlated well with baicalin and molecules that were related to fever, we established a novel network pharmacology study and the results no only validated the antipyretic activity of baicalin and elucidated its antipyretic mechanisms as well.

A notable aspect of this strategy is the determination of target tissue, here, hypothalamus was chosen as the target tissue of QKLI for its antipyretic effect. It is worldwide accepted that fever is an important brain-mediated response and the preoptic region of the anterior hypothalamus is the major thermoregulatory centre in the central nervous system (CNS), containing larger numbers of thermosensitive neurons that are responsible for the reception and integration of the temperature signals generated by the periphery and centre^23–26^. In addition, there are also many heat mediums in this region, such as corticotrophin-releasing factor (CRF), NO, cAMP, PGE2 and so on. Thus, the hypothalamus is thought to be the target tissue of febrile reaction and was chosen as the target tissue to study the antipyretic effect QKLI.

As we know, there are two pathways for fever signal transduction, the humoral and neural pathways^27–29^. In previous work, we always focused on exploring the humoral mechanisms of antipyretic effect of QKLI. Many works have been done to investigate the metabolic profile in the urine and plasma of yeast-induced pyrexia rats after QKLI administration and found the potential biomarkers in order to explore the antipyretic mechanisms of QKLI. The urine metabolomics identified that the antipyretic effect of QKLI on yeast-induced pyrexia rats was performed by repairing the perturbed metabolism of amino acids^30^. Plasma study was also performed and the result showed that the antipyretic mechanism of QKLI was performed by correcting the perturbed pathways of amino acid metabolism and lipid metabolism^31^. In addition, plasma pharmacokinetics study of QKLI demonstrated that baicalin and geniposide might be the potential antipyretic active ingredients of QKLI^32^. This works could be helpful for revealing the antipyretic active components and antipyretic mechanisms of QKLI to some degree. However, since the hypothalamus is the target tissue of fever, the treatment of fever drugs should pass through the BBB to regulate certain receptors in the CNS for producing effect. Exactly what components of QKLI can pass through the BBB and reach its target tissue did not have detailed reports. Thus, in this study, an integrated strategy was established based on target tissue analysis for rapid identifying the antipyretic components and mechanisms of QKLI.

According to this strategy, baicalin was identified as the antipyretic component of QKLI and its antipyretic mechanisms were elucidated as well. In previous studies, the antipyretic effect of baicalin have been proved by both *in vitro* and *in vivo* researches^33, 34^, this confirms from another aspect that the strategy developed in this study is feasible for screening the active components of TCM. It is notable that, although only baicalin was screened as the antipyretic component of QKLI in this study, it alone cannot represent the QKLI for the whole antipyretic activity. As we mentioned above, there are two pathways for fever signal transduction, the humoral and neural pathways, and the present study just have explored the neural mechanisms of QKLI for its antipyretic effect. There may be some other antipyretic components in QKLI that can through the humoral pathways to exert their antipyretic effect, this need to be validated in future researches.

**Figure 8:**
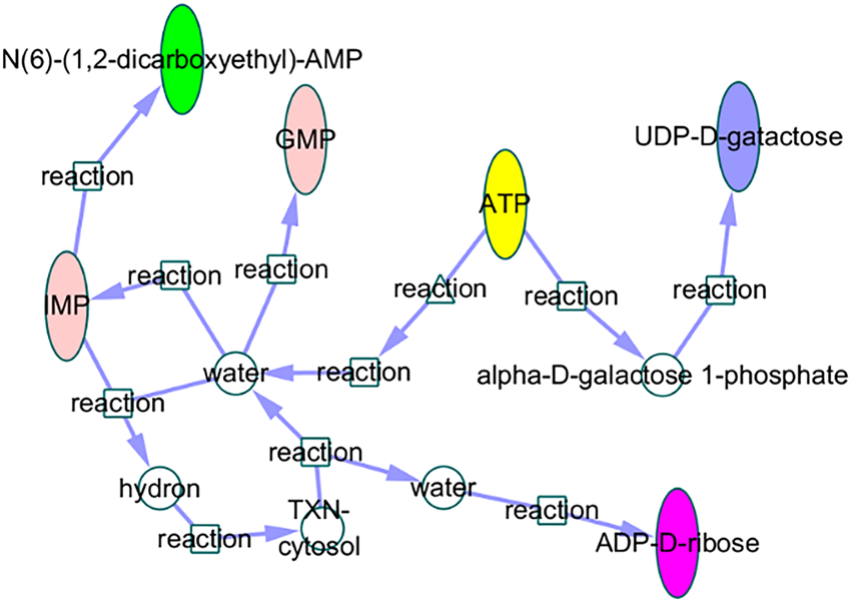
Metabolic pathways of ATP and the verified biomarkers (Yellow indicates ATP, blue indicates UDP-D-galactose, rose red indicates ADP–D-ribose, pink indicates IMP and GMP and green indicates N6-(1,2-dicarboxyethyl)-AMP).

According to previous researches, it indicated that baicalin could obstruct the increase of PGE2 and cAMP in the hypothalamus to act its antipyretic effect^35^.The targets of baicalin such as HIF-1α could relate to PGE2 and cAMP by a series of reactions and metabolic pathways in the constructed metabolic networks. Thus, we can speculate that baicalin may decrease the concentration of PGE2 and cAMP by inhibiting the expression of HIF-1α or other important targets in this pathway and then exert antipyretic effect. Besides, there were also lots of researches proved the regulation of PGE2 to fever, and other molecule such as NO and AA, played a specific role in the fever response^15^. Another target of baicalin, CASP3, could synthesize NO by some reactions, thus indirectly inducing fever. So we inferred that baicalin could inhibit the express of CASP3 or other important targets in the pathways then inhibited the increase of NO to relieve fever. In addition, AA played a key role in the synthesis of PGE2 and PGF2α, metabolic AA could relate to metabolic pathways of many inflammations, and inflammations were closely linked with fever. Therefore, baicalin could inhibit the synthesis of AA so that inhibited the increase of PGE2. It was reported that extracellular purine nucleotides and nucleosides signaling molecules, such as ATP and adenosine, play important roles in regulating fever-related molecules by targeting on P2 receptor, which exists in hypothalamic and brainstem nuclei, are involved in the body temperature regulation process. Besides, as shown in Fig. 8, ATP is the critical node of multiple pathways and the five biomarkers of QKLI could all track back to ATP through some pathways, thus proved the reliability of the networks. In summary, we could speculate the fever metabolic pathways mediated by baicalin and clarify its antipyretic mechanisms, but we could not determine the specific target of baicalin in the hypothalamus. Therefore, it still required further experimental studies to resolve the problem.

In conclusion, we have proposed an integrated strategy by using target tissue metabolomics biomarkers as pharmacodynamic surrogate indices for identifying the active components and mechanisms of TCM. The application of this strategy to QKLI allowed us to identify baicalin as the antipyretic components and elucidate its antipyretic mechanisms, this gave a better understand of the therapeutic effect of QKLI. Besides, this efficient, practical, and universally applicable strategy can also be extended to identify active components and mechanisms for other complex drugs besides TCM.

## Methods

### Chemicals and materials

QKLI was obtained from Shineway Pharmaceutical Co., Ltd (Hebei, China). The reference standards of baicalin, geniposide and isorhamnetin (internal standard, IS; purity >98% for each compound) were purchased from the National Institute for the Control of Pharmaceutical and Biological Products (Beijing, China). Yeast was acquired from Mauri Food Co., Ltd (Hebei, China). LC/MS-grade methanol and acetonitrile were supplied by Fisher Scientific (Fair Lawn, NJ, USA), HPLC-grade formic acid was obtained by ROE Scientific (Newcastle, Delaware, USA). Ultra high purity water was generated from the Synergy UV water purification system (Millipore Corp., Billerica, MA, USA). The composition of artificial cerebrospinal fluid (ACSF) was as follows: NaCl 145.0 mmol/L; KCl 2.7 mmol/L; CaCl_2_ 1.2 mmol/L; MgCl_2_ 1.0 mmol/L; NaH_2_PO_4_ 0.45 mmol/L; Na_2_HPO_4_ 1.55 mmol/L; pH 7.4.

### Animals

Experiments were conducted on 84 SPF-grade male Sprague-Dawley rats weighting 280 ± 20 g, which were purchased from Bijing Weitonglihua Laboratory Animal Technology Co., Ltd (Beijing, China). Before use in the study, all the rats were kept under controlled environmental conditions (temperature, 20 ± 2 °C; relative humidity, 60 ± 5%; 12 h dark- light cycle) with free access to food and water for one week. Each animal was used only once. All animal experiments were approved by the Animal Ethics Committee of the Beijing University of Chinese Medicine (Beijing, China), and all procedures were performed in accordance with the Helsinki Declaration.

### Apparatus

Ultra performance Liquid Chromatogram combined with triple-quadrupole tandem mass spectrometer, equipped with an electrospray ionization source (Waters corp. USA); Thermo Accela™ HPLC-LTQ/Orbitrap coupled LC–MS system (Thermo Fisher Scientific, Bremen, Germany); Markerlynx 4.1 software (Waters corp. USA); Microdialysis system including MAB microdialysis probe (MAB Corp. Sweden), ALS-IP800L microdialysis pump (Biological Technology Co., Shanghai), stereotaxis instrument, refrigerated fraction collectors (MAB Corp. Sweden).

### Study protocol

#### Experiment I: Hypothalamus microdialysis study of Qingkailing injection in CG and MG rats

A total of 12 SPF-grade male Sprague-Dawley rats (280 ± 20 g) were selected and divided into two groups randomly, the CG and MG. For hypothalamus microdialysate collection, each rat was fixed to the stereotaxic apparatus after anesthesia (10% chloral hydrate, 3.5 mL/Kg). The MAB probe wire was embedded in the hypothalamus by microdialysis operation (the specific process of operation was described in Supplementary Information). Subsequently, microdialysis probes were inserted into the rats’ hypothalamus under anesthesia with chloral hydrate after one day’s recovery. The MG rats were injected with a 40% aqueous suspension of yeast (7.5 mL/kg) in the back below the nape. The CG rats were similarly given an equal volume of 0.9% saline. Four hours after molding, microdialysis probes were perfused with ACSF at a constant flow rate of 1.5 μL/min. Probes were allowed to equilibrate for 60 min before beginning sample collection. Five hours after molding^36^, rats were administered via the tail vein of 8 mL/kg QKLI. Then the dialysis sample was collected from each animal at every 30 min for 5 h.

#### Experiment II: Hypothalamus microdialysis study of Qingkailing injection and hypothalamus metabolomic profiling

In this part, experiments were conducted on 72 SPF-grade male Sprague-Dawley rats (TG rats) weighing 280 ± 20 g, and these rats were randomly assigned to CG (n = 6), MG (n = 6) and TG (N = 60, divided into 10 groups for different time point group (0.5 h, 1 h, 1.5 h, 2 h, 2.5 h, 3 h, 3.5 h, 4 h, 4.5 h, and 5 h after QKLI administration)). For hypothalamus microdialysate collection, rats were treated under protocols in accordance with Experiment I. After microdialysis probes were inserted into the rats’ hypothalamus under anesthesia with chloral hydrate after one day’s recovery, the MG and TG rats were injected with a 40% aqueous suspension of yeast (7.5 mL/kg) in the back below the nape, the CG rats were similarly given an equal volume of 0.9% saline. Four hours after molding, microdialysis probes were perfused with ACSF at a constant flow rate of 1.5 μL/min. Probes were allowed to equilibrate for 60 min before beginning sample collection. Five hours after molding^36^, the CG and MG rats were sacrificed for the hypothalamus samples collection, while the TG rats were administered via the tail vein of 8 mL/kg QKLI. Then the single dialysis sample was collected at 0.5 h, 1 h, 1.5 h, 2 h, 2.5 h, 3 h, 3.5 h, 4 h, 4.5 h, and 5 h from different time point group rats. Immediately after hypothalamus microdialysate collection, the TG rats were sacrificed for the hypothalamus samples collection.

For hypothalamus samples collection, all animals were sacrificed by decapitation and the brains removed immediately. The entire hypothalamus was dissected from the brain and then the hypothalami were excised. The total dissection time was < 2 min, and the hypothalami were washed with physiological saline, and then weighed precisely after being dried with filter paper. Finally, the hypothalamus samples were stored at −80 °C until analysis.

### Determination of baicalin and geniposide concentrations in the hypothalamus microdialysate by UPLC-MS/MS

The concentrations of baicalin and geniposide were determined by UPLC-MS/MS. Prior to analysis, 10 μL of the IS working solution (295 ng/mL in ACSF) was added to 40 μL of dialysates. Chromatographic separation was performed on an Acquity UPLC BEH C18 column (100 × 2.1 mm i.d., 1.7 μm; Waters Corp., Milford, MA, USA). The column temperature was maintained at 40 °C and the autosampler was conditioned at 4 °C. The mobile phase was composed of A (0.1% formic acid in water) and B (acetonitrile) at a flow rate of 0.4 mL/min in a run time of 5 min. Gradient condition of the mobile phase was as follows: 0–0.5 min maintained at 1% B; 0.5–2 min, increased from 1% B to 25% B; 2–4 min, increased from 25% B to 50% B; 4–4.5 min, increased from 50% B to 57% B; 4.5–4.6 min, isocratic 1% B; 4.6–5 min, maintained at 1% B for column equilibrium. The injection volume was 5 μL.

The mass spectrometer was operated in the negative ion mode by multiple reaction monitoring (MRM) of the transition of *m/z* 445.15 → 269.01 for baicalin, *m/z* 433.15 → 225.01 for geniposide, and *m/z* 315.16 → 300.14 for IS. Optimal MS parameters were as follows: capillary voltage 3.0 kV; cone voltage 30 V; Nitrogen was used as the desolvation gas and cone gas with the flow rate at 800 and 150 L/h, respectively. Argon was used as collision gas at a pressure of approximately 3.4 × 10^−3^ mbar. Temperature of the source and desolvation set at 150 and 400 °C, respectively. The optimized cone voltage and collision energy were 38 V and 22 eV for baicalin, 38 V and 14 eV for geniposide and 64 V and 20 eV for IS, respectively.

### Hypothalamus metabolomics study

Hypothalamus sample preparation for HPLC-LTQ/Orbitrap MS analysis was processed according to our previously published work^16^. 50 mg hypothalamus tissue samples were mixed with 500 μL chilled methanol/water (4:1, v:v), homogenized in an ice bath and then were deproteinised by centrifugation at 4 °C (14,000 rpm, 10 min). The supernatant was evaporated to dryness under a gentle stream of nitrogen and reconstituted with methanol/water (1:1, v:v) for HPLC-LTQ/Orbitrap MS analysis.

Separation of metabolites was performed on a Thermo Accela™ HPLC-LTQ/Orbitrap coupled LC–MS system (Thermo Fisher Scientific, Bremen, Germany). For chromatographic separation, a Diamonsil C18 column (4.6 × 250 mm, 5 μm) was used. The mobile phase was composed of A (0.05% formic acid in water) and B (acetonitrile) with a linear gradient elution: 0–5 min, maintained at 2% B; 5–15 min, increased from 2% B to 15% B; 15–20 min, increased from 15% B to 40% B; 20–30 min, increased from 40% B to 57% B; 30–45 min, increased from 57% B to 72% B; 45–60 min, increased from 72% B to 100% B; 60–61 min, isocratic 2% B; 61–70 min, maintained at 2% B for column equilibrium. The flow rate was 1 mL/min. The autosampler was maintained at a temperature of 4 °C, and 10 μL of sample solution was injected for each run.

The mass spectrometer was operated in both positive and negative ion mode. Data were collected from *m/z* 50 to *m/z* 1000. The source parameters in positive (and negative) mode were as follows: heater temperature of 300 °C, sheath gas flow rate of 30 arb, auxiliary gas flow rate of 10 arb, I spray voltage of 4 (3) kV, capillary voltage of 25 (−35) V, and tube lens of 110 (−110) V.

### Data Analysis

Phoenix WinNonlin Ver.6.3 program was applied in detecting the concentration of the components of QKLI in the hypothalamic dialysates of noncompartmental model fitting pharmacokinetic parameters. The HPLC-LTQ/Orbitrap MS raw data were transformed using TransOmics™ to obtain a three-dimensional data matrix containing the retention time, *m/z*, and peak intensity of each sample, and then the data matrix was introduced to Metaboanalysis 3.0 for PCA and PLS-DA analysis. MS fragments were extracted using Xcalibur software and combined with some available biochemical databases, such as METLIN (http://metlin.scripps.edu/), HMDB (http://www.Hmdb.ca/), and KEGG (http://www.kegg.com) to identify metabolites. The regression analysis between potential biomarkers and the concentration of baicalin and geniposide was accomplished using the SPSS16.0 software.

### Network pharmacology analysis

See Supplementary Information for details on each step of network pharmacology analysis.

## Electronic supplementary material

Supporting Information
