# Supplementary material for: An integrated strategy by using target tissue metabolomics biomarkers as pharmacodynamic surrogate indices to screen antipyretic components of Qingkaikling injection
Source: Sci Rep. 2017 Jul 24;7:6310. doi: 10.1038/s41598-017-05812-0 (PMC5524955; doi:10.1038/s41598-017-05812-0)
Supplement: Supplementary file 1 — Supporting Information [file 41598_2017_5812_MOESM1_ESM.doc]

**Supporting Information**

**An integrated strategy by using target tissue metabolomics biomarkers as pharmacodynamic surrogate indices to screen antipyretic components of Qingkaikling injection**

Zhixin Zhang^1, a^, Fang Lu^2, a^, Haiyu Liu^1^, Huizhen Zhao^1^, Yuehong Liu^1^, Shuang Fu^1^, Meiling Wang^1^, Ziye Xie^1^, Honghong Yu^1^, Zhenghai Huang^1^, Yanling Zhang^2^, * & Xiaoyan Gao^1^, *

^1^ School of Chinese Material Medica, Beijing University of Chinese Medicine, South of Wangjing Middle Ring Road, Chaoyang District, Beijing 100102, PR. China

^2^ Key Laboratory of TCM Foundation and New Drug Research, School of Chinese Material Medica, Beijing University of Chinese Medicine, South of Wangjing Middle Ring Road, Chaoyang District, Beijing 100102, PR. China

^a^ Co-first author

*Correspondence and requests for materials should be addressed to Y.-L.Z. (email: zhangyanling@bucm.edu.cn) or X.-Y.G. (email: [gaoxiaoyan0913@sina.com](mailto:gaoxiaoyan0913@sina.com)).

**1. Hypothalamus microdialysis study**

**Microdialysis surgery**

Male Sprague-Dawley rats were fixed on stereotaxis instrument after anesthesia (10% chloral hydrate, 3.5 mL/Kg). We slited the scalp, explored the outcrop and drilled a hole (1 mm) at the location of AP-1.5 mm, ML-3.2 mm, DV-5.2 mm, according to the rats’ brain localization mapping^1^. MAB probe wire was embedded in the hypothalamus of rats and fixed on the cranium with screw and denture acrylic and then the skin was sutured. The rats were fed with standard laboratory chow with water *ad libitum* for one day and then were performing molding experiment.

**Preparation of standard and quality control (QC) samples**

The stock solutions of baicalin, geniposide and IS were freshly prepared in methanol individually. The combined standard working solutions of these analytes were prepared by mixing standard solutions with artificial cerebrospinal fluid (ACSF). The IS solution was brought to a final concentration of 295 ng/mL in ACSF. Calibration standard solutions (baicalin concentrations: 0.312, 0.780, 1.560, 3.120, 7.800, 15.600, 31.200, 78, 156 and 312 ng/mL; geniposide concentrations: 0.270, 0.675, 1.350, 2.700, 6.750, 13.500, 27, 67.500, 135, 270 ng/mL.) were prepared in ACSF prior to each analytical run. QC samples, which were used in the validation, were prepared from stock solutions by independent dilution at three levels for baicalin 0.624, 15.600 and 280.800 ng/mL; 0.540, 13.500, 243 ng/mL for geniposide. 10 μL of the IS was added to 40 μL of working solution before UPLC-MS/MS analysis.

**Determination of microdialysis probe recovery rate**

Counter-dialysis method was used for *in vivo* recovery determinations^2^. The recovered rats were anesthetized by 10% chloral hydrate (3.5 mL/Kg) through intraperitoneal injection and fixed. MAB probe was inserted into the probe wick. ACSF was poured into the probe at the rate of 1.5 μL/min and this process lasted for 1 h. Then ACSF with concentrations of baicailin 15.6 ng/mL and geniposide 13.5 ng/mL was used to balance the system for 30 min. Dialysate was collected for 10 times with 45 μL each time and was analyzed by UPLC-MS/MS. The injection volume was 5 μL. The relative recovery was estimated calculated by the equation: Rin vivo = (1-C_dialysate_/C_perfusate_) ×100%^3^. C_dialysate_ represented the average concentration of dialysate while C_perfusate_ represented the concentration of perfusate.

**Results**

**Recovery of microdialysate**

The recoveries of microdialysate were investigated and the results indicated that baicalin recovery was 48.33 ± 1.03% and geniposide recovery was 45.63 ± 0.61%.

**Selectivity**

The selectivity of the method was tested by comparing the chromatograms of blank microdialysates (n = 6) and the blank microdialysates spiked with the analytes and IS, and microdialysis samples obtained after intravenous administration of qingkailing injection (QKLI). All blank microdialysates lots were found to be free of interferences with the compounds of interest. Under the above conditions the retention time of baicalin, geniposide and IS was 3.4, 2.6 and 4.2 min, respectively (Figure S1).

**Figure S1. Multiple reaction monitoring chromatograms for analytes and IS in rat hypothalamus microdialysis samples: (A) a blank blank microdialysates sample; (B) a blank microdialysates sample spiked with the two components and IS; (C) a microdialysates sample from a rat at 0.5 h after a single intravenous administration of qingkailing injection (QKLI).**

**Sensitivity and linearity**

The lower limit of quantitation (LLOQ) of the baicaillin and geniposide, defined as the lowest concentration on the standard curve that can be quantitated with accuracy within 20% of nominal and precision not exceeding 20% CV, was 0.312 ng/ml and 0.270 ng/ml. The LLOQ was determined by examining six LLOQ samples independent from the standard curve.

Calibration curves were constructed by plotting the peak area ratios of microdialysates standards *vs.* nominal concentration. The calibration model was selected based on the analysis of the data by linear regression with/without intercepts and weighting factors (1/x, 1/x^2^ and none). The best linear fit and least-squares residuals for the calibration curve were achieved with a 1/x weighting factor, giving a mean linear regression equation for the calibration curve of baicalin: y = 25.255x + 4.24413, r^2^ = 0.9991 and geniposide : y = 12.0461x + 3.57623, r^2^ = 0.9998, where y represents the peak area ratios of the two analytes to that of IS, and x represents the concentration of two analytes in ng/ml. Calibration curves were linear in the range 0.312-312 ng/ml for baicalin and 0.270-270 ng/ml for geniposide, with r^2^ ≥ 0.999.

**Accuracy and precision**

The method showed good accuracy and precision. Table S1 shows a summary of intra- and inter-day accuracy and precision for baicaillin and geniposide from the QC samples, respectively. In this assay, the intra-day precision was less than 4.242%, the inter-day precision was less than 6.476%. The accuracy ranged from -6.173% to 3.917%.

**Table S1. Accuracy and precision of the analytes in rat microdialysates samples (n = 18, 6 replicates per day for three days)**

| Compound | Concent  (ng/mL) | Intra-day (n=6) | | | Inter-day (n=18) | | |
| --- | --- | --- | --- | --- | --- | --- | --- |
|  |  | Concentration measured  (ng/mL) | Precision  (%, RSD) | Accuracy  (%, RE) | Concentration measured  (ng/mL) | Precision  (%, RSD) | Concentration  (%, RE) |
| baicalin | 0.624 | 0.641 ± 0.024 | 3.759 | 2.698 | 0.648 ± 0.025 | 3.812 | 3.917 |
|  | 15.600 | 15.634 ± 0.347 | 2.219 | 0.219 | 15.677 ± 0.297 | 1.895 | 0.491 |
|  | 280.800 | 279.859 ± 16.544 | 5.912 | -0.335 | 283.753 ± 12.036 | 6.476 | 1.052 |
| Geniposide | 0.540 | 0.507 ± 0.033 | 4.242 | -6.173 | 0.525 ± 0.039 | 1.895 | -2.829 |
|  | 13.500 | 13.965 ± 0.249 | 1.786 | 3.449 | 13.821 ± 0.222 | 1.603 | 2.378 |
|  | 243.00 | 248.076 ± 7.522 | 3.032 | 2.089 | 242.795 ± 9.326 | 3.841 | -0.008 |

**Matrix effect**

In this study, the matrix effects were evaluated by comparing the peak areas of samples spiked with those of standard solutions at the same concentration. The matrix effects derived from QC samples were in the range of 93.256%～107.419% (Table **S**2) , showing that the ACSF matrix effect could be negligible under the current conditions.

**Table S2. Matrix effect of the analytes in rat microdialysates samples (n=6)**

| Compound | Concentration (ng/mL) | Matrix effects (%) |
| --- | --- | --- |
| Baicalin | 0.624 | 103.700 |
|  | 15.600 | 107.419 |
|  | 280.800 | 98.269 |
| Geniposide | 0.540 | 106.841 |
|  | 13.500 | 90.963 |
|  | 243.000 | 93.256 |

**Stability**

Stability studies were conducted at three QC levels with three replicates in different conditions that occurred during sample analysis. The short-term stability was evaluated with QC samples stored at room temperature for 4 h. The long-term stability was assessed by QC samples kept at –80°C for 7 days. The post-preparative stability was measured by determining QC samples maintained in the autosampler conditions at 4°C for 12 h.The stabilities of all analytes at different conditions are shown in Table S3. The analytes were found to be stable in the microdialysates samples when stored at room temperature for 4 h, at –80°C for 7 days. Post-preparative samples were also stable when kept in the autosampler at 4°C for 12 h.

**Table S3. Accuracy and precision of the analytes in microdialysates samples (n = 18, 6 replicates per day for three days)**

| Compound | Concentration (ng/mL) | Stabality (RE%) | | |
| --- | --- | --- | --- | --- |
|  |  | Post-treatment  12 h in autosampler | Short-term  4 h at room temperature | Long-term  7 days at –80°C |
| Baicalin | 0.624 | 10.209 | 2.967 | -1.335 |
|  | 15.6 | 8.735 | 4.688 | -1.038 |
|  | 280.8 | 2.408 | 2.345 | 0.762 |
| Geniposide | 0.54 | -4.136 | 1.173 | -11.605 |
|  | 13.5 | 0.457 | 0.447 | -5.405 |
|  | 243 | 2.316 | 0.656 | -3.202 |

**2. Hypothalamus metabolomics study**

Figures S2 shows the typical total ion chromatograms (TIC) from CG, MG, and TG rats at different sampling time points (0.5 h, 1 h, 1.5 h, 2 h, 2.5 h, 3 h, 3.5 h, 4 h, 4.5 h and 5 h).

**CG**

**MG**

**0.5 h**

**1 h**

**1.5 h**

**2 h**

**A**

**2.5 h**

**3 h**

**3.5 h**

**4 h**

**4.5 h**

**5 h**

**B**

**CG**

**MG**

**0.5 h**

**1 h**

**1.5 h**

**2 h**

**2.5 h**

**3 h**

**3.5 h**

**4 h**

**4.5 h**

**5 h**

**Figures S2. TIC chromatograms of CG, MG, and TG rats at different sampling time points derived from HPLC-LTQ/Orbitrap MS analysis. (A) Positive ion mode. (B) Negative ion mode.**

**Validation of the HPLC-LTQ/Orbitrap MS conditions**

A quality control (QC) sample was produced by mixing equal aliquots (50 mg) of each hypothalamus sample. The precision was evaluated by injecting one QC sample six times. The reproducibility was determined by the analysing six replicates of QC samples on one day. The system stability test was carried out by injecting a QC samples every 10 samples during the entire sample analysis procedure.

Extracted ion chromatographic peaks of five ions with retention times and *m/z* pairs of 2.74–148.0598, 12.59–269.0869, 23.40–318.2987, 36.14–437.1912, and 44.74–533.3524 were selected for the method validation in the positive ion mode. The relative standard deviations (RSDs) of the retention time for the precision, reproducibility and system stability were found to be 0.07–0.75%, 0.05–1.19%, and 0.36–1.73%, respectively, while the RSDs for peak area were within the ranges of 1.34–5.52%, 2.31–7.74% and 0.54–4.69%. The relative errors (REs) of the peak area for the post-preparative stability were found to be -0.68–7.57%.

In the negative ion mode, five ions with retention times and *m/z* pairs of 10.54–362.0478, 12.22–267.0717, 21.96–326.942, 41.20–480.3083, and 43.20–540.3273 were selected. The RSDs of the retention time for the precision, reproducibility and system stability were found to be 0.03–1.36%, 0.05–1.05%, and 0.06–1.24%, while the RSDs for the peak area were within the ranges of 1.31–5.63%, 0.89–2.42%, and 1.37–7.62%. The REs of the peak area for the post-preparative stability were found to be -0.82–3.68%. These results indicated that the developed method showed good precision, reproducibility and stability for hypothalamus samples analysis.

**Identification of potential biomarkers**

The mass data information were obtained from the HPLC-LTQ/Orbitrap MS and then combined with information from online databases such as METLIN, and HMDB to identify the potential biomarkers. Here, we selected the 364.0648 *m/z* to describe the procedure of structural identification in detail. First, the accurate mass of the ion was extracted using the Xcalibur workstation (shown in Figure S3A). The fragments were also gained as shown in Figure S3B and C. Second, the METLIN database was searched. The ion was obtained in positive ion mode, so it was possible to generate the [M+H]^+^ ion. GMP was identified as the most probable biomarkers. The mass information obtained using the METLIN was also consistent with the information extracted from the Xcalibur. To prove the rationality of the result, the fragments were analysed. GMP lost the pentose phosphate to yield a fragment of *m/z* 151.8742, it was simply guanosine. The ion of *m/z* 151.8742 lost 17 to generate a fragment of *m/z* 134.8327, indicating that the metabolite contains a group of -NH_2_. Therefore, the metabolite was ultimately identified as GMP. Other metabolites were identified following the same procedure.

**Figure S3. Spectra of ion fragment in MS^n^ analysis of GMP. (A) MS^1^, (B) MS^2^, (C) MS^3^**.

**3. Network pharmacology analysis**

A metabolism network of baicalin was constructed and utilized to illustrate the potential antipyretic mechanism of baicalin at molecular level. First, two metabolism networks about the targets of baicalin and fever-related small molecules were constructed respectively. Second, in order to mediate the relationship between baicalin and fever-related molecules, a network of the verified biomarkers was constructed. Meanwhile, to ensure the final metabolism network of baicalin has good connectivity, another metabolism network was constructed based on the targets of the verified biomarkers. Third, the final metabolism network of baicalin was obtained by merging the above four networks together. The Specific strategy of this paper was as Figure S4.

**Construction of baicalin targets network**

The targets of baicalin were derived from the STITCH Database (http://stitch.embl.de/). To ensure the reliability of results, the parameter of the required confidence score was set higher than 0.7. Then, the metabolic pathways of these targets were collected from the Reactome Pathway Database (http://www.reactome.org/). After that, the metabolic pathways were merged together and the duplicated edges were removed by using Cytoscape 3.2.1. Thus, the network of baicalin-related targets was obtained and was named as network A.

**Construction of fever-related molecules network**

Fever-related molecules, such as prostaglandin E2 (PGE2), cyclin adenosine monophosphate (cAMP), nitric oxide (NO), arachidonic acid (AA), and prostaglandin F2alpha (PGF2α), were collected from related references^4-6^. The metabolic pathways of them were obtained from Reactome Database. Then, the network of fever-related molecules was constructed by using the same method as described above and was defined as network B.

**Construction of biomarkers network**

By entering the CHEBI IDs of the verified biomarkers (“15919”, “17345”, “18307”, “16960”, and “17202”) as search terms in the Reactome Database, the corresponding metabolic pathways were obtained. Then, the network of biomarkers was constructed by using the same method as described above and was defined as network C.

**Construction of biomarkers-related targets network**

Five biomarkers, including N6-(1,2-dicarboxyethyl)-AMP, GMP, UDP-D-galactose, ADP-D-ribose, and IMP, were verified in the experiment. By adopting the same parameter in STITCH Database as described above, the biomarkers-related targets were obtained. Then the metabolic pathways of these targets were derived from the Reactome Database. Following, the network of biomarkers-related targets was constructed by using the same method as described above and was defined as network D.

**Merging network A, B, C, and D together**

The constructed network A, B, C, and D were merged together and the duplicated edges were removed by using Cytoscape3.2.1. Thus, the final metabolism network of baicalin was obtained. To illuminate the direct antipyretic effects of baicalin, the start node of the network was set using baicalin-related targets and the end node of the network was set using the fever-related molecules. The verified biomarkers and their related targets were contained in the pathways which connected the start node and the end node in the network.


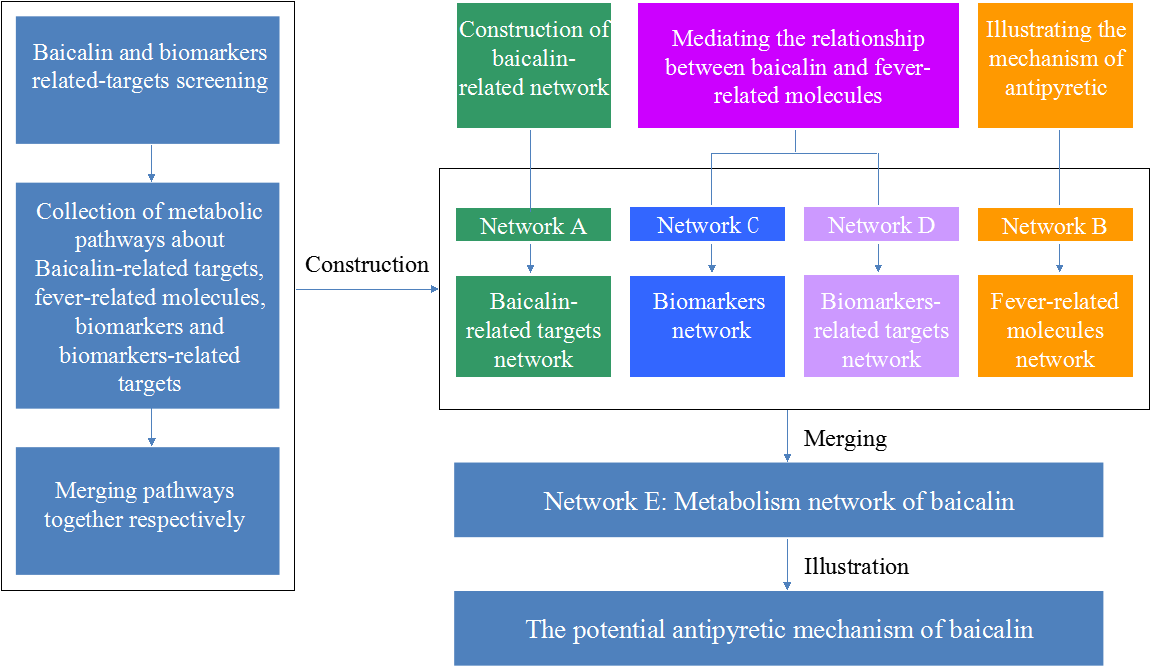


**Figure S4. The strategy of metabolism network of baicalin constructed.**

**References**

1. Bao, X. M. et al. The rat brain in stereotazic coordinates 1st edition. *Beijing: People’s Medical Publishing House* 27-46 (1991).

2. Song, W. T. et al. Application of micodialysis technique in medicine field. *China Journal of Chinese Materia Medica* **34** 247-250 (2009).

3. Hsiao, J. K. et al. Effects of different semipermeable membranes on in vitro and in vivo performance of microdialysis probes. *J Neurochem* **54** 1449-1452 (1990).

4. Soares, D. M. et al. CCL3/macrophage inflammatory protein-1alpha induces fever andincreases prostaglandin E2 in cerebrospinal fluid of rats: effect of antipyreticdrugs. *Brain Res* **1109** 83–92 (2006).

5. Reis, R. C. et al. Central substance P NK_1_ receptors are involved in fever induced by LPS but not by IL-1β andCCL3/MIP-1α in rats. *Brain Res* **1384** 161–169 (2011).

6. Guo, M. X. et al. Characterization of rational biomarkers accompanying fever in yeast-induced pyrexia rats using urine metabolic footprint analysis. *J Pharm Biomed Anal* **95** 68–75 (2014).
